# Emission of single photons in the weak coupling regime of the Jaynes Cummings model

**DOI:** 10.1038/s41598-020-72945-0

**Published:** 2020-09-30

**Authors:** Changsuk Noh

**Affiliations:** Department of Physics, Kyungpook National University, Daegu, 41566 Korea

**Keywords:** Quantum physics, Single photons and quantum effects

## Abstract

A recently proposed variant of an unconventional photon blockade scheme is studied for a single emitter weakly coupled to a resonator mode. By controlling two weak coherent fields driving the emitter and the resonator mode, a strongly nonclassical output field is obtained, which is not only antibunched, but has vanishing higher photon number coincidences. For a given set of system parameters, the frequencies and strengths of the driving fields that yield such an output are given.

## Introduction

Producing nonclassical light, especially single photons, is an important goal in quantum optics with applications in quantum technologies^1–4^. In cavity QED systems single photons can be produced through the so-called photon blockade effect^5^, in which a strong coupling between an emitter and a cavity mode yields an effective photon-photon interaction that prevents occupation of multiple photons in the cavity mode^6,7^. Photon blockade is characterized by vanishing second-order correlation functions at zero delay, and have been observed in many physical systems^8–11^. It is generally associated with the so-called strong coupling regime, but actually can be observed in weak coupling regimes^12^ just like other nonclassical features^13,14^.

More recently, it has been found that photon blockade can be observed for arbitrarily small coupling strengths by carefully tuning parameters in a two-cavity system^15^. The system under investigation was a coupled weakly Kerr-nonlinear oscillators where individually the nonlinearity was not large enough to induce photon blockade. Soon after, the cause of the phenomenon was identified to be quantum interference, and it was further shown that only one of the oscillators needs to be nonlinear and that the effect persists if the nonlinearity is provided by coupling a two-level atom to the oscillator^16^. This inspired interference-based phenomena in similar setups involving two cavity modes and a quantum dot^17,18^ and led to experimental demonstrations^19,20^. Generalizations such as asymmetric losses^21^, cavities with second order susceptibility^22^, with two driving fields^23–25^, under pulsed excitation^26^ have also been investigated. Effects of mixing the output channels have also been investigated^27^ and the antibunching obtained by interfering a nonclassical and a classical states of light has been shown to be related to UPB^28^.

In Ref.^29^, it was discovered that the fine-tuning can be relegated to an additional driving field. The experimentally difficult task of fine-tuning the required system parameters can therefore be bypassed, making it much easier to produce antibunched photons with weakly nonlinear cavities. The purpose of this work is to investigate the scheme in detail for an emitter–resonator system described by the Jaynes–Cummings model. The emitted photons are found to be not only antibunched, but have suppressed higher-order photon coincidences to all order. This feature therefore goes beyond UPB, which was shown to be equivalent to optimizing a Gaussian state to suppress the two-photon coincidences^30^, with enhanced probabilities for higher photon numbers^29^. This means that photons are emitted one-by-one. The cause of this behaviour can be attributed to the strong nonlinearity in the two-level emitter, as will be explained throughout this work.

### Model

The Jaynes–Cummings Hamiltonian reads

1 $$\begin{aligned} H_{\mathrm{JC}} = \omega _r a^\dagger a + \omega _0 \sigma _+\sigma _- + g\left( a\sigma _+ + a^\dagger \sigma _-\right) , \end{aligned}$$

which describes a resonator with a natural frequency $\omega _r$ coupled to a two-level emitter with a transition frequency $\omega _0$. If both the resonator mode and emitter are driven with monochromatic fields of frequencies $\omega _p$ and $\omega _d$ respectively, it becomes

2 $$\begin{aligned} H = H_{\mathrm{JC}} + \Omega_p^* e^{i\omega _p t} a + \Omega _p e^{-i\omega _p t} a^\dagger + \Omega _d^* e^{i\omega _d t} \sigma _- + \Omega _d e^{-i\omega _d t} \sigma _+. \end{aligned}$$

Going into the frame rotating at the probe frequency $\omega _p$, the Hamiltonian changes to

3 $$\begin{aligned} H =&\Delta _r a^\dagger a + \Delta _0 \sigma _+\sigma _- + g\left( a\sigma _+ + a^\dagger \sigma _-\right) \nonumber \\&+\Omega _p( a + a^\dagger ) + \Omega _d^* e^{i\Delta _d t} \sigma _- + \Omega _d e^{-i\Delta _d t}\sigma _+, \end{aligned}$$

in which $\Delta _r = \omega _r - \omega _p$, $\Delta _0 = \omega _0 - \omega _p$ and $\Delta _d = \omega _d - \omega _p$. For $\omega _p = \omega _d$, the Hamiltonian becomes time independent:

4 $$\begin{aligned} H = \Delta _r a^\dagger a + \Delta _0 \sigma _+\sigma _- + g\left( a\sigma _+ + a^\dagger \sigma _-\right) +\Omega _p( a + a^\dagger ) + \Omega _d^* \sigma _- + \Omega _d \sigma _+. \end{aligned}$$

This case was briefly studied in^29^ showing that, surprisingly, $\Omega _d$ can always be tuned to obtain UPB given an arbitrary $\Omega _p$.

To see this, consider the quantum master equation, which includes dissipations in the emitter and resonator modes:

5 $$\begin{aligned} {\dot{\rho }} = -i[H,\rho ] + {\mathcal {D}}[\sqrt{\kappa }a]\rho + {\mathcal {D}}[\sqrt{\gamma }\sigma _-]\rho , \end{aligned}$$

in which

6 $$\begin{aligned} {\mathcal {D}}[c]\rho = c\rho c^\dagger - \frac{1}{2}c^\dagger c \rho - \frac{1}{2}\rho c^\dagger c. \end{aligned}$$

$\kappa$ and $\gamma$ denote dissipation rates for the resonator and atom, respectively. Next, approximating the dissipative part by ignoring the $c\rho c^\dagger$ parts of the dissipators, the above master equation reduces to the Schrödinger equation with a non-Hermitian Hamiltonian,

7 $$\begin{aligned} H_{\mathrm{nH}}= H -i\frac{\kappa }{2}a^\dagger a - i\frac{\gamma }{2} \sigma _+ \sigma _-, \end{aligned}$$

which amounts to making changes $\Delta _r \rightarrow \Delta _r -i \kappa /2 \equiv {\tilde{\Delta }}_r$ and $\Delta _0 \rightarrow \Delta _0 -i \gamma /2 \equiv {\tilde{\Delta }}_0$ in Eq. (4)

Now let us label the state as $|n,\alpha \rangle$, in which *n* indicates the number of excitations in the resonator mode and $\alpha = \{ g,e \}$ denotes the ground and excited state of the atom, respectively. Then to a low number of excitations, a general state is given by

8 $$\begin{aligned} |\psi (t) \rangle&= c_{0g}|0,g\rangle + c_{1g}|1,g\rangle + c_{0e}|0,e\rangle + c_{2g}|2,g\rangle + c_{1e}|1,e\rangle + \cdots . \end{aligned}$$

In this basis, ignoring the contributions from the states with a higher number of excitations, the equations of motion are

9 $$\begin{aligned} i{\dot{c}}_{0g}&= \Omega ^*_p c_{1g} + \Omega ^*_d c_{0e}, \nonumber \\ i{\dot{c}}_{1g}&= \Omega _p c_{0g} + g c_{0e}+ {\tilde{\Delta }}_r c_{1g} + \sqrt{2} \Omega ^*_r c_{2g} + \Omega ^*_d c_{1e}, \nonumber \\ i{\dot{c}}_{0e}&= \Omega _d c_{0g} + g c_{1g}+ {\tilde{\Delta }}_0 c_{0e} + \Omega ^*_p c_{1e}, \nonumber \\ i{\dot{c}}_{2g}&= \sqrt{2}\Omega _p c_{1g} + \sqrt{2}g c_{1e}+ 2{\tilde{\Delta }}_r c_{2g}, \nonumber \\ i{\dot{c}}_{1e}&= \Omega _p c_{0e} + \sqrt{2}g c_{2g} + \Omega _d c_{1g} + {\tilde{\Delta }}c_{1,e}, \end{aligned}$$

in which $${\tilde{\Delta }} \equiv {\tilde{\Delta }}_{r}+ {\tilde{\Delta }}_{0}$$.

To solve for the steady state, one assumes $c_{0g} \approx 1$ and solves for $c_{1g}$ and $c_{0e}$ ignoring the contributions from higher number of excitations^13^. This yields

10 $$\begin{aligned} c_{1g} \approx \frac{g\Omega _d - {\tilde{\Delta }}_0\Omega _p}{{\tilde{\Delta }}_0{\tilde{\Delta }}_r - g^2}, \;\; c_{0e} \approx \frac{g\Omega _p - {\tilde{\Delta }}_r\Omega _d}{{\tilde{\Delta }}_0{\tilde{\Delta }}_r - g^2}, \end{aligned}$$

which can be substituted into the last two equations of motion to give

11 $$\begin{aligned} c_{1e}&\approx \frac{\Omega _d\Omega _p(g^2+{\tilde{\Delta }}_r^2+{\tilde{\Delta }}_r{\tilde{\Delta }}_0) - g(\Omega _p^2{\tilde{\Delta }}+\Omega _d^2{\tilde{\Delta }}_r)}{({\tilde{\Delta }}_0{\tilde{\Delta }}_r - g^2)({\tilde{\Delta }}{\tilde{\Delta }}_r - g^2)}, \nonumber \\ c_{2g}&\approx \frac{{\tilde{\Delta }}\Omega _p({\tilde{\Delta }}_0\Omega _r - 2g\Omega _d)+g^2(\Omega _p^2+\Omega _d^2)}{\sqrt{2}({\tilde{\Delta }}_0{\tilde{\Delta }}_r - g^2)({\tilde{\Delta }}{\tilde{\Delta }}_r - g^2)}. \end{aligned}$$

The condition for vanishing two-photon excitation, $c_{2g} = 0$, is then approximated by

12 $$\begin{aligned} g^2\Omega _d^2 -2g {\tilde{\Delta }}\Omega _p \Omega _d + \Omega _p^2({\tilde{\Delta }}{\tilde{\Delta }}_0+g^2) \approx 0. \end{aligned}$$

The solutions

13 $$\begin{aligned} \Omega _{d}^{\pm } = \frac{\Omega _p}{g}\left( {\tilde{\Delta } \pm {\text{sgn}}(\Omega _p)\sqrt{{\tilde{\Delta }}{\tilde{\Delta }}_r - g^2}}\right) \end{aligned}$$

thus give two values of $\Omega _d$ for which the two photon correlation function $g^{(2)}(0)$ vanishes. This condition, first derived in^29^, shows that a low value of $g^{(2)}(0)$ can be achieved in the weakly-driven regime if one carefully tunes the amplitude and phase of the field driving the atom.

### Single driving field

Let us first consider the case in which $\Omega _d = 0$. In this case, antibunching can only be observed if both the coupling strength and one of the detunings are chosen carefully. From (12), one readily sees that these are

14 $$\begin{aligned} \Delta _{0}^{\mathrm{opt}} = -\frac{\gamma \Delta _r}{\kappa +2\gamma }, \quad g^{\mathrm{opt}} = \pm \sqrt{\frac{\gamma \Delta _r^2(\kappa +\gamma )}{(\kappa +2\gamma )^2} + \frac{(\kappa +\gamma )\gamma }{4}}. \end{aligned}$$

With these parameters, destructive interference occurs between the pathways to reach $|2,g\rangle$, resulting in an almost-vanishing second order correlation function

15 $$\begin{aligned} g^{(2)}(0) = \frac{\langle a^\dagger a^\dagger a a \rangle }{\langle a^\dagger a \rangle ^2}. \end{aligned}$$

For example, with $\Delta _r = 0$, $\Omega _p = 0.01$, and $\kappa = \gamma = 1$, we have $\Delta _{0}^{\mathrm{opt}} = 0$ and $g^{\mathrm{opt}} = 1/\sqrt{2}$, giving $g^{(2)}(0) \approx 0.003$ in the steady state with the average photon number ${\bar{n}} \approx 4\times 10^{-5}$. These and all numerical results in this work are obtained by numerically solving Eq. (5) using QuTiP^31^. For a larger value of detuning $\Delta _r$, the required value of $g^{\mathrm{opt}}$ becomes larger as can be clearly seen from Eq. (14).

In^30^, UPB was shown to be related to Gaussian states: the fine tuning of the parameters correspond to choosing optimal values of squeezing in a generic Gaussian state. Because of this Gaussianity, the suppression from the Poisson photon number distribution is limited to the two-photon manifold for UPB and comes at the cost of enhanced probabilities for higher photon numbers^29^. This means that the output field in the UPB scheme is *n*-photon bunched for $n\ge 3$, i.e. $g^{(n)}(0) = \langle a^{\dagger n} a^{n}\rangle /\langle a^\dagger a \rangle ^n > 1$. Similar behaviour is observed for the current weakly coupled case with a single driving field. For the above parameters, $g^{(n)}(0) = 13, 158, 903$ for $n = 3,4,5$ respectively. Introducing the atom-driving field changes this—the higher photon number manifolds are also suppressed.

### Two driving fields

**Figure 1:**
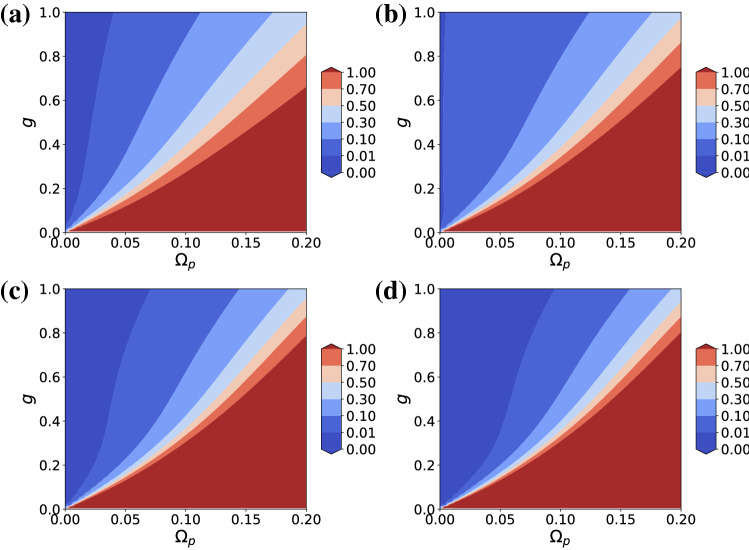
Contour plots of (**a**) $g^{(2)}(0)$, (**b**) $g^{(3)}(0)$, (**c**) $g^{(4)}(0)$, and (**d**) $g^{(5)}(0)$ as functions of *g* and $\Omega _p$ for $\Delta = 0$ and $\omega _p = \omega _-$. All units are in terms of $\kappa = \gamma = 1$.

With two driving fields, it is no longer necessary to tune the ‘internal’ values *g* and $\Delta _0$. The addition of 2 more control parameters is enough to guarantee vanishing second order correlation function even in the weak coupling regime $g \lesssim \kappa , \gamma$. Figure 1a shows the contour plot of $g^{(2)}(0)$ in the steady state as a function of *g* and $\Omega _p$. The driving frequency is fixed to the first excited state (assuming $\omega _r \ge \omega _0$) of the JC model, $\omega _- = (\omega _r+\omega _0 - \sqrt{\Delta ^2+4g^2})/2$, with $\Delta = \omega _r - \omega _0$ and will remain so unless otherwise stated. $\Omega _d = \Omega _d^+$ is chosen for reasons that will be explained below. The result makes sense intuitively. The larger the value of *g* the larger the nonlinearity, which in turn allows for a stronger drive strength until nonclassicality is lost. In other words, the strength of the driving field is limited by the value of the coupling strength, which sets the nonlinearity of the system. Unlike in the UPB scheme, higher order correlations are also suppressed as shown in Fig. 1b–d. Three photon coincidence as given by $g^{(3)}(0)$ has a smaller region of suppression compared to $g^{(2)}(0)$, but higher order correlations are suppressed in wider regions. Higher order $g^{(n)}(0)$’s not shown are also suppressed. For weak driving strengths as considered here, average photon number ${\bar{n}} \ll 1$ and the chance of observing *n*-photons decreases exponentially with *n*, but in view of possible generalization to single photon applications it is nice to have suppressed coincidence counting too all orders.

The fact that merely adding another driving field can make such a big change to all orders of correlation functions may seem surprising, but one should note that the additional driving field addresses the highly non-linear two-level emitter as emphasized in^11^. In fact, in the bad cavity regime characterized by $\kappa \gg g^2/\kappa \gg \gamma$, the emitter-driven system is commonly used as a single-photon source^2^. One can therefore understand the suppressed occurrences of two or more photons as resulting from a combination of non-classical light produced by driving the emitter and UPB which further suppresses the two-photon coincidence. To see this, let us consider the case in which only the atom is driven, i.e. $\Omega _p = 0$ and $\Omega _d = 0.01$. This simplification allows one to obtain an analytic solution for higher-order correlation functions in a manageable form. For a given *n* the equations of motion (9) generalizes to (still ignoring the contributions from the higher number excitations)

16 $$\begin{aligned} i{\dot{c}}_{ng}&\approx \sqrt{n}gc_{n-1e} + n{\tilde{\Delta }}_r c_{ng}, \nonumber \\ i{\dot{c}}_{n-1e}&\approx \sqrt{n}gc_{ng} + \left( (n-1){\tilde{\Delta }}_r +{\tilde{\Delta }}_0\right) c_{n-1e} + \Omega _dc_{n-1g}. \end{aligned}$$

In the steady state the solutions are

17 $$\begin{aligned} c_{n-1e} \approx -\frac{\sqrt{n}{\tilde{\Delta }}_r}{g}c_{ng}, \quad c_{ng} \approx \frac{g\Omega _d}{\sqrt{n}\left[ {\tilde{\Delta }}_r\left( (n-1){\tilde{\Delta }}_r +{\tilde{\Delta }}_0 \right) -g^2\right] }c_{n-1g}. \end{aligned}$$

Repeatedly applying the second equation to itself, one obtains

18 $$\begin{aligned} c_{ng}&\approx \prod _{j=1}^n \frac{g\Omega _d}{\sqrt{n+1-j}\left[ {\tilde{\Delta }}_r\left( (n-j){\tilde{\Delta }}_r +{\tilde{\Delta }}_0 \right) -g^2\right] } \nonumber \\&= \prod _{j=0}^{n-1} \frac{g\Omega _d}{\sqrt{j+1}\left[ {\tilde{\Delta }}_r\left( j{\tilde{\Delta }}_r +{\tilde{\Delta }}_0 \right) -g^2\right] } \nonumber \\&= \frac{g^n\Omega _d^n}{\sqrt{n!}} \prod _{j=0}^{n-1} \frac{1}{\left[ {\tilde{\Delta }}_r\left( j{\tilde{\Delta }}_r +{\tilde{\Delta }}_0 \right) -g^2\right] }. \end{aligned}$$

Using Eq. (10), *n*th order correlation function at zero delay can thus be approximated by

19 $$\begin{aligned} g^{(n)}(0) \approx \frac{n!|c_{ng}|^2}{|c_{1g}|^{2n}} \approx \prod _{j=0}^{n-1} \frac{ \left| {\tilde{\Delta }}_0{\tilde{\Delta }}_r-g^2\right| ^{2}}{\left| {\tilde{\Delta }}_r\left( j{\tilde{\Delta }}_r +{\tilde{\Delta }}_0 \right) -g^2\right| ^2} . \end{aligned}$$

We get $\Delta _r = \Delta _0 = -g$ for $\Delta = 0$ and assuming $g \ll \gamma ,\kappa$, the above equation can be further simplified to

20 $$\begin{aligned} g^{(n)}(0) \approx \prod _{j=0}^{n-1} \frac{ \left( -\frac{\gamma \kappa }{4}\right) ^{2}}{\left| -j\frac{\kappa ^2}{4} -\frac{\gamma \kappa }{4}\right| ^2} = \prod _{j=0}^{n-1} \frac{\gamma ^2\kappa ^2}{ \kappa ^2 \left( \gamma +j\kappa \right) ^2} = \prod _{j=0}^{n-1} \frac{\gamma ^2}{ \left( \gamma +j\kappa \right) ^2}. \end{aligned}$$

For $\gamma =\kappa$, $g^{(n)}(0) \approx \prod _{j=0}^{n-1} 1/(1+j)^2$. This formula yields $g^{(2)}(0) \approx 1/4$, $g^{(3)}(0) \approx 1/36 \approx 0.027$, and $g^{(4)}(0) \approx 1/576 \approx 0.0017$ for the set of parameters used in Fig. 1, except for $\Omega _d = 0.01$ and $\Omega _p = 0$. These agree reasonably well with the numerically obtained results $g^{(2)}(0) = 0.26$, $g^{(3)}(0) = 0.03$, and $g^{(4)}(0) =0.002$, for *g* = 0.1, where the differences are due to the terms ignored in going from Eqs. (19) to (20). The above formula shows that higher-order correlations are well-suppressed for $n>2$ when the atom is driven and the atom-field coupling is weak, but that antibunching is not strong.

**Figure 2:**
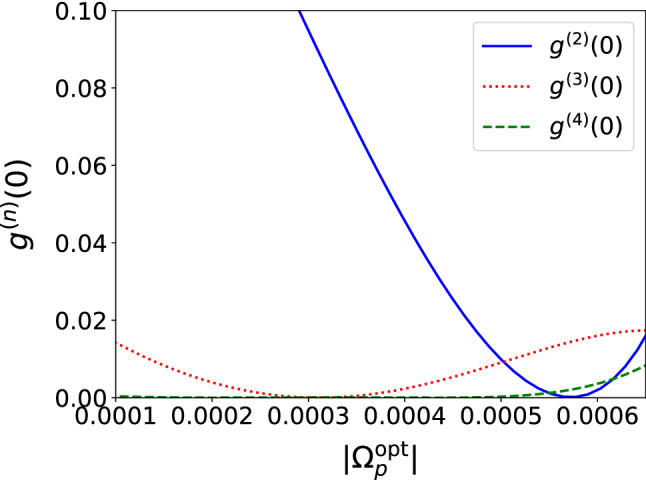
$g^{(n)}(0)$ for $n=2,3,4$ as functions of $|\Omega _p^{\mathrm{opt}}|$. *g* = 0.1, $\Delta = 0$, $\omega _p = \omega _-$ and $\Omega _d = 0.01$. The phase of $\Omega _p$ has been set to its optimal value and only the overall magnitude is varied. All units are in terms of $\kappa = \gamma = 1$.

Upon increasing $\Omega _p^{\mathrm{opt}}$, which is to be obtained from Eq. (12) after allowing $\Omega _p$ to be complex, a numerical calculation shows that $g^{(2)}(0)$ varies rapidly while higher-order correlations do not, as illustrated in Fig. 2. This shows that one can obtain good suppression of all *n*th order correlation functions for *n* ≥ 2, by controlling the magnitude of $\Omega _p$. We can therefore understand the single-photon behaviour of the emitted photons as resulting from a combination of non-classical light produced by driving the emitter, and UPB which suppresses the two-photon coincidence.

### Strength of the atom-driving field

In Eq. (13), there were two choices for $\Omega _d$. So far, $\Omega _d^+$ has been chosen, but what if $\Omega _d^-$ were used instead? It turns out that all correlation functions become larger in the parameter regimes considered above. For example, for $g = 0.1$, $\Omega _p= 0.01$, $g^{(n)}(0) = (0.145, 14.2, 200, 1580)$ is obtained instead of $(0.022, 0.026, 0.005, 3.50\times 10^{-4})$ for $n=2,3,4,5$. The difference is in the magnitude of $\Omega _d$. For our choice of the resonator-driving frequency $\omega _p = \omega _-$, $\vert \Omega _d^+\vert > \vert \Omega _d^-\vert$ and it is evidently advantageous to drive the emitter more strongly. If $\omega _p$ were chosen to be $\omega _+ = (\omega _r+\omega _0 + \sqrt{\Delta ^2+4g^2})/2$, such that the second excited state is driven, the situation is reversed. The magnitudes of $\Omega _d^\pm$ are merely reversed although their phases are changed. The values $g^{(n)}(0) = (0.145, 14.2, 200, 1580)$ are obtained for $\Omega _d = \Omega _d^+$, and the lower values are obtained for $\Omega _d = \Omega _d^-$. Therefore, $\omega _p$ can be chosen to be either $\omega _+$ or $\omega _-$ as long as the correct $\Omega _d$ is used.

This freedom only holds as long as the atomic transition is resonant to the cavity mode, i.e. $\omega _r = \omega _0$. If this were not the case, as in many experimental implementations, merely choosing the larger of $\Omega _d^\pm$ turns out to be insufficient. In addition, the correct resonator-driving frequency must be chosen, which in this case (Δ > 0) is $\omega _-$. For example, with $\omega _p = \omega _+$, $\omega _r = 12$ and the rest of the parameters remaining the same, neither $\Omega _d^+$ nor $\Omega _d^-$ yields single-photon correlations. For $\Omega _d = \Omega _d^-$, $g^{(n)}(0) = (0.068, 0.90, 2.06, 10.0)$ for $n=2,3,4,5$ while for $\Omega _d = \Omega _d^+$, $g^{(n)}(0) = (0.107, 11.9, 160, 1470)$. This asymmetry between $\omega _-$ and $\omega _+$ can be understood by noting that for $\Delta = \omega _r - \omega _0 \ge 0$ the first excited state is predominantly in $|0,e\rangle$, which is the preferred state to be driven (because we want to drive the atom, not the cavity). On the other hand, if $\Delta < 0$, then the situation is reversed and the privileged role is taken by the second excited state.

**Figure 3:**
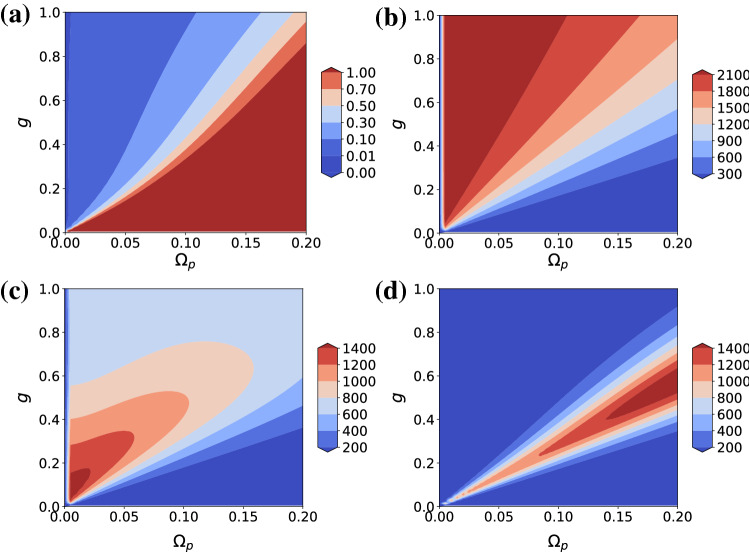
Contour plots of $\vert \vert (g^{(k)})\vert \vert _4$ for (**a**) $(\omega _-,\Omega _d^+)$, (**b**) $(\omega _-,\Omega _d^-)$, (**c**) $(\omega _+,\Omega _d^+)$, and (**d**) $(\omega _+,\Omega _d^-)$ as functions of *g* and $\Omega _p$ for $\Delta = 2$. All units are in terms of $\kappa = \gamma = 1$.

To quantify the suppression of higher-order photon coincidences let us adopt a measure called $n-norm$, defined as $\vert \vert (g^{(k)})\vert \vert _n = \root n \of {\sum _{k=2}^{n+1}[ g^{(k)} ]}$^32^. It measures the distance in the correlation space between the given source and an ideal single-photon source. $4-norm$s are adopted in this work, but higher-order *norms* are also suppressed. Figure 3a–d show that out of the four different combinations with $\omega _p = \omega _\pm$ and $\Omega _d = \Omega _d^\pm$, the combination $(\omega _-,\Omega _d^+)$ gives the best result as explained above. It is not shown but $g^{(2)}(0)$ is always suppressed and the corresponding figures are qualitatively similar to Fig. 1a.

**Figure 4:**
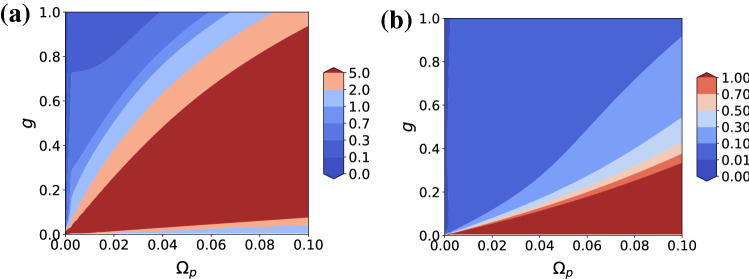
Contour plots of $\vert \vert (g^{(k)})\vert \vert _4$ for (**a**) $\Delta = 2$ and (**b**) $\Delta = 0$ as functions of *g* and $\Omega _p$. $\omega _p = \omega _+$ and $\Omega _d = \Omega _d^-$. All units are in terms of $\kappa = \gamma = 1$. Note the differences in the contour values.

Figure 4a gives a close-up view of Fig. 3d, which shows that $(\omega _+,\Omega _d^-)$*can* in fact yield good single-photon characteristics in a limited region of the parameter space. Note the changes in the range of $\Omega _p$ and the color coding. However, the figure shows that it is very difficult to obtain good single-photon characteristics for $g \ll \gamma , \kappa$. Figure 4b is the corresponding figure for $\omega _r = \omega _0$, showing a much wider region of suppressed $\vert \vert (g^{(k)})\vert \vert _4$. It is exactly the same as the one obtained using $(\omega _-,\Omega _d^+)$. From these facts we see that non-zero detuning breaks the symmetry between $(\omega _+,\Omega _d^-)$ and $(\omega _-,\Omega _d^+)$, which can again be explained by the relative fraction of $|0,e\rangle$ in the addressed state.

To sum up, to obtain good single-photon characteristics one must: (1) choose the driving frequency such that the state $|0,e\rangle$ is predominantly driven and (2) choose the driving strength such that the emitter-driving field is the stronger of $\Omega _\pm$ in Eq. (13). Then the strong nonlinearity of the atom guarantees that the photons are emitted one by one in a large region of parameter space. If a ‘wrong’ choice is made, two-photon coincidences are still suppressed but higher-order ones are not, as in UPB.

**Figure 5:**
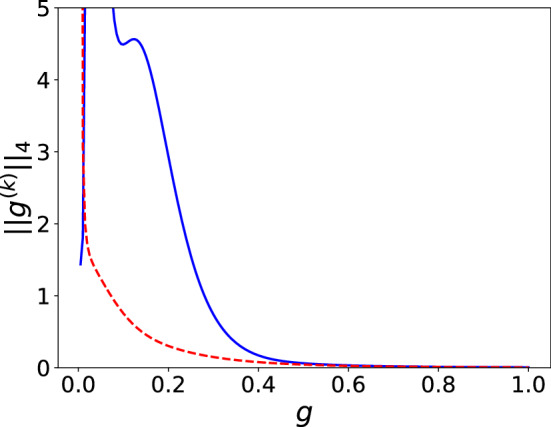
$4-norm$s as functions of *g* for $\Omega _p = 0.01$ (solid blue curve) and $\Omega _p = 0.001$ (dashed red curve). $\Delta = 0$, $\kappa = 0.1$, and $\gamma = 1$.

Lastly, let us briefly consider asymmetries in $\kappa$ and $\gamma$. The bad-cavity regime characterized by $\kappa \gg g^2/\kappa \gg \gamma$ is often invoked for the purpose of creating single photons. The emitter is driven, which mainly emits into the leaky cavity mode. Even in this regime, additional cavity-driving field improves the quality of output single photons. For example, for $\kappa = 1, g = 0.1, \gamma = 0.01, \omega _r = \omega _0 = 10$, $\omega _p = \omega _-$, and $\vert \Omega _d \vert \approx 0.11$, the first three values of $g^{(n)}(0)$ are (0.10, 0.0032, 0.000046) for $\Omega _p = 0$ and (0.0079, 0.0012, 0.000062) for $\Omega _p = 0.01$ (obtained from $\Omega _d^+$ in Eq. (13)). On the other hand, if the atom decays much faster, i.e., $\gamma \gg g,\kappa$ the driving field strengths need to be reduced and the suppression for higher order photon coincidences are not so good. For $\gamma = 1, \kappa = g = 0.1$ and $\Omega _p = 0.01$, the first three values of $g^{(n)}(0)$ are (0.19,0.82,1.2), which reduces to (0.0019,0.54,0.25) for $\Omega _p = 0.001$. Figure 5 shows how $\vert \vert g^{(n)} \vert \vert _4$ changes as a function of *g* for $\Omega _p = 0.01$ (solid blue curve) and $\Omega _p = 0.001$ (dashed red curve). The values differ significantly for $g<0.5$. For $\Omega _p = 0.01$ it is interesting that $\vert \vert g^{(k)} \vert \vert _4$ gets bigger first before it decreases, indicating strong multi-photon bunching.

In summary, a generalization of a scheme based on UPB proposed recently in^29^ has been investigated in detail. It is shown that unlike in UPB, the output field has suppressed probabilities for all *n*-photon coincidences for $n \ge 2$. The main reason for the suppression can be attributed to the strong nonlinearity of the 2-lv system, upon which the interference effects of UPB are added to reduce the two-photon coincidences further. One aspect not discussed in this work is the time-variation of the correlation functions. In the conventional UPB scenario, the coupling strength *J* between the resonators need to be large for weak nonlinearities. This causes the second order correlation function to oscillate rapidly on the time scale of order 1/*J*. Such rapid oscillations are absent in the system investigated in this work, because there is no large parameter involved. That the correlation functions change on the time scale of $1/\gamma$ and $1/\kappa$ has been checked by numerical calculations.

As a future work it would be interesting to investigate whether the proposed scheme can be generalized to produce an on-demand single photon source which typically requires a multi-level emitter. Due to the small coupling strength required and the ease of parameter tuning, such a scheme would prove to be very useful.
